# Microstructured Magnetoactive Elastomers for Switchable Wettability

**DOI:** 10.3390/polym14183883

**Published:** 2022-09-17

**Authors:** Raphael Kriegl, Gaia Kravanja, Luka Hribar, Lucija Čoga, Irena Drevenšek-Olenik, Matija Jezeršek, Mitjan Kalin, Mikhail Shamonin

**Affiliations:** 1 East Bavarian Centre for Intelligent Materials (EBACIM), Ostbayerische Technische Hochschule (OTH) Regensburg, Seybothstr. 2, 93053 Regensburg, Germany; 2 Laboratory for Laser Techniques, Faculty of Mechanical Engineering, University of Ljubljana, Aškerčeva 6, SI-1000 Ljubljana, Slovenia; 3 Laboratory for Tribology and Interface Nanotechnology, Faculty of Mechanical Engineering, University of Ljubljana, Bogišićeva 8, SI-1000 Ljubljana, Slovenia; 4 Faculty of Mathematics and Physics, University of Ljubljana, Jadranska 19, SI-1000 Ljubljana, Slovenia; 5 Department of Complex Matter, J. Stefan Institute, Jamova 39, SI-1000 Ljubljana, Slovenia

**Keywords:** magnetoactive elastomer, wetting, contact angle, surface microstructuring, laser micromachining, optical profilometry

## Abstract

We demonstrate the control of wettability of non-structured and microstructured magnetoactive elastomers (MAEs) by magnetic field. The synthesized composite materials have a concentration of carbonyl iron particles of 75 wt.% (≈27 vol.%) and three different stiffnesses of the elastomer matrix. A new method of fabrication of MAE coatings on plastic substrates is presented, which allows one to enhance the response of the apparent contact angle to the magnetic field by exposing the particle-enriched side of MAEs to water. A magnetic field is not applied during crosslinking. The highest variation of the contact angle from (113 ± 1)° in zero field up to (156 ± 2)° at about 400 mT is achieved in the MAE sample with the softest matrix. Several lamellar and pillared MAE structures are fabricated by laser micromachining. The lateral dimension of surface structures is about 50 µm and the depth varies between 3 µm and 60 µm. A systematic investigation of the effects of parameters of laser processing (laser power and the number of passages of the laser beam) on the wetting behavior of these structures in the absence and presence of a magnetic field is performed. In particular, strong anisotropy of the wetting behavior of lamellar structures is observed. The results are qualitatively discussed in the framework of the Wenzel and Cassie–Baxter models. Finally, directions of further research on magnetically controlled wettability of microstructured MAE surfaces are outlined. The obtained results may be useful for the development of magnetically controlled smart surfaces for droplet-based microfluidics.

## 1. Introduction

In recent years, materials with stimuli-responsive surface properties have gained significant attention in the scientific community [1,2,3,4,5,6,7]. Such “smart” surfaces with stimuli-responsive changes in wettability are of interest for the development of, for example, micro- and nanofluidic devices, self-cleaning and anti-fog surfaces and sensor devices [8,9,10,11].

The wetting properties of solid substrates can be controlled by external stimuli such as light [12,13], heat [14,15], pH-value [16] and electric [17,18] or magnetic fields [6,7,19,20,21]. These surfaces can also be structured (possess a particular topography, for example, pillars), e.g., on the micrometer scale. The advantage of stimuli-responsive surfaces is that they can dynamically change their properties according to the time-varying stimulus. Among different external stimuli, dynamic control of magneto-responsive surfaces (MRSs) has been shown to be particularly useful, because it can be achieved by off-the-shelf permanent magnets, which do not require constant power supply. Alternatively, the regulation can be conveniently achieved by compact electromagnets. These MRSs can have many intriguing functionalities such as remote, non-invasive and rapidly tunable liquid manipulation. Consequently, they find important applications in various areas, e.g., droplet-based microfluidics [21,22,23], liquid transporters/distributors [22,24,25], fog harvesters [26] and soft-robot locomotion [25,27,28].

In the present paper, we explore the so-called magnetoactive elastomers (MAEs), which can be classified as hybrid materials [29] comprising micro- and nanometer-sized magnetic particles (inorganic constituent) embedded into a soft polymer matrix (organic constituent) [30]. MAEs are multifunctional composite materials whose physical properties (elastic moduli, electrical conductivity, magnetic and dielectric properties, etc.) can significantly change in technically easily realizable magnetic fields (magnetic flux density *B* ≲ 400 mT). It is the softness of the polymer matrix that enables large changes of physical properties in a magnetic field. In such a matrix, the embedded ferromagnetic particles change their mutual arrangement (microstructure) in an external magnetic field in order to align themselves along magnetic field lines. This phenomenon is called restructuring of the filler [31].

**Figure 1:**
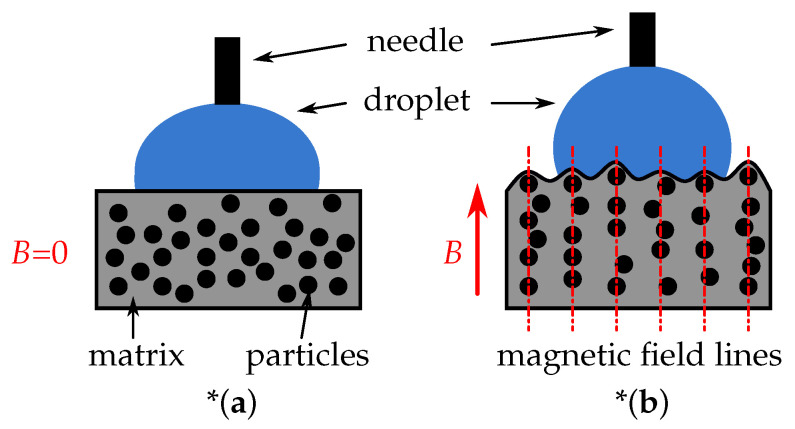
Schematic drawings of magnetic field-induced modifications of surface morphology of MAEs. The red arrow indicates the field direction. (**a**) Initial (“smooth”) MAE surface. (**b**) Increased surface roughness due to magnetic-field-induced restructuring of the filler due to the rearrangement of filler particles. *Note that magnetic particles are attached to the matrix. Therefore, the compliant polymer matrix becomes deformed in the magnetic field [58]. These internal deformations of the matrix are not depicted in the Figure.

The state of the art of research and development of MAEs is well documented in several reviews [30,31,32,33,34,35,36,37,38,39,40,41,42], where the overwhelming majority of the results refer to the bulk properties. It has been recently realized that MAEs are very promising materials for rapid and reversible control of various surface properties, in particular, wettability [23,43,44,45], surface roughness (SR) [44,46], adhesion [47] and friction properties [48]. To date, it is understood that in soft MAEs with Young’s modulus below 100 kPa (in the absence of a magnetic field), a magnetic field applied in the direction perpendicular to the surface induces the formation of “mountain-like” structures that resemble the Rosensweig peaks in ferrofluids [49] and produce a strong increase in SR [46,50,51,52,53,54,55,56]. Figure 1 illustrates the underlying physical effect. In our recent characterizations of this effect, we found that the sensitivity of root mean square (rms) roughness Sq to magnetic field has an order of magnitude from 1–10 µm/T [44,57]. It is commonly believed that the increase in SR is responsible for the increased hydrophobicity. It has been shown that MAE’s SR in the absence of a magnetic field can be influenced by crosslinking in the presence of a magnetic field [23]. Hitherto, we are aware of only four papers [23,43,45,52] that have considered magnetically regulable wettability of unstructured MAE surfaces in experiments. Furthermore, the magnetic control of wettability of prestructured MAE-based MRSs remains essentially unexplored. Very recently, a laser-based facile method for structuring of MAE surfaces on the µm scale has been proposed [21]. However, it remains unclear how the laser ablation affects the wettability of such microstructured MAE surfaces, both in the absence and in the presence of a magnetic field.

The purpose of this paper is twofold. First, we present a novel scalable method for fabricating large-area MAE films with enhanced response of hydrophobicity to an applied magnetic field. Second, we investigate in detail the wettability of MAE films made with different stiffnesses of the elastomeric matrix and with different parameters of laser micro-structuring on the MAE surfaces, both in the absence and in the presence of a magnetic field. To simplify the analysis of results obtained, we investigated the switchability of MAE surfaces, which is characterized by a significant difference of a property of interest in the activated state (a magnetic field of ≈400 mT is switched on) compared to the deactivated state (absence of the field).

## 2. Materials and Methods

### 2.1. Synthesis of MAE Materials

The synthesis of MAE samples followed the principles described by us in [59,60]. The base polymer VS 100,000 (vinyl-functional polydimethylsiloxane, PDMS) for addition-curing silicones, the chain extender modifier 715 (SiH-terminated PDMS), the reactive diluent polymer MV 2000 (monovinyl functional PDMS), the crosslinker (CL) 210 (dimethyl siloxane-methyl hydrogen siloxane copolymer), the Pt-Catalyst 510 and the divinyl-tetra-methyldisiloxane (DVS) inhibitor were provided by Evonik Hanse GmbH, Geesthacht, Germany. The silicone oil WACKER® AK10 (linear, non-reactive PDMS) was purchased from Wacker Chemie AG, Burghausen, Germany. The soft-magnetic carbonyl iron powder (CIP) type SQ (mean particle size d50 of 4.5 µm, no coating), provided by BASF SE Carbonyl Iron Powder & Metal Systems (Ludwigshafen, Germany), was used as the ferromagnetic filler. The polymer VS 100,000, the polymer MV 2000, the modifier 715 and the silicone oil AK 10 were put together and blended with an electric hand mixer to form an initial compound. In the next step, the initial compound was mixed with CIP and crosslinker 210. The crosslinking (hydrosilylation) reaction was activated by the Pt-Catalyst 510. For activity control of the Pt-Catalyst, an inhibitor was employed. A vacuum stirrer to remove trapped gas bubbles was used. All samples had the same CIP concentration of ≈75 wt.% (≈27 vol.%). This particular volume fraction of iron particles was used because the highest magneto-mechanical coupling as manifested by the MR effect should be expected there [61,62]. Moreover, at typical volume fractions of CIP (≈22–33 vol.%), the softness of the material is expected to be more important than the concentration of the filler for efficient restructuring of the magnetic filler on an MAE surface [52]. The elastomer was cured in an air-circulated oven at 80 °C for 1h followed by 60 °C for 24 h.

Table 1 summarizes chemical compositions of fabricated MAEs. Table 2 presents the resulting mechanical properties of synthesized composites as the mean value ± standard deviation. It can be seen that the initial shear storage modulus of the composite can be modified by changing the crosslinker content because it directly affects the ratio of molar concentrations of vinyl and hydride groups in the PDMS matrix [63]. In the following, the MAE materials are denoted as “soft” (S), “medium” (M) and “hard” (H) according to their relative stiffness.

### 2.2. New Method for Fabrication of MAE Surfaces

**Figure 2:**
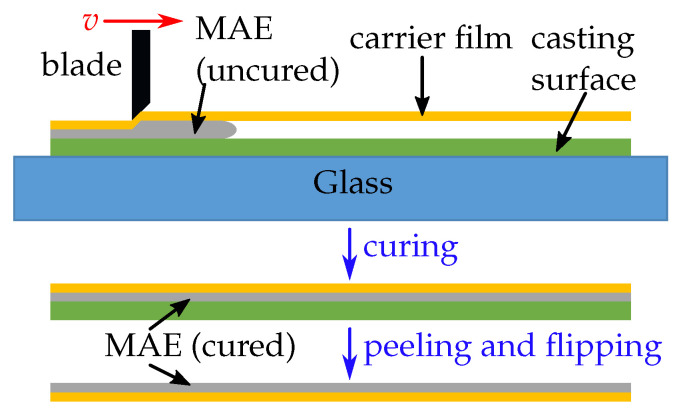
“Upside-down” fabrication method for an MAE film on a plastic carrier film.

To enhance the desired response of MAE-based MRSs, a new fabrication method was developed. As seen in Figure 2, the mixture was poured onto a casting surface, in our case a polystyrene (PS) plate. This was chosen because it allows easy removal of the cured MAE film. After the uncured, viscous MAE mixture was poured over the casting surface, a plastic (polyethylene terephthalate, PET) carrier film with a thickness of 0.1 mm was cut to size (approximately 100 × 200 mm ${}^{2}$) and placed over the slurry (our realization of this new method allows for samples up to 210 × 297 mm ${}^{2}$ in area). This assembly was then put into a film applicator (TQC Sheen Automatic Film Applicator Compact, Industrial Physics, New Castle, DE, USA), where a blade (TQC Sheen Micrometric film applicator 100 mm, Industrial Physics, New Castle, DE, USA) moves atop the PET foil with a constant speed of *v* = 1 mm/s and spreads the mixture between the foil and the surface. The resulting sample thickness can be varied by adjusting the blade distance via micrometer screws. The assembly is put into the oven for the curing process, which is described above. After the composite has been cured, the PET-foil and the MAE film stick together and can be peeled off the casting plate. For wetting experiments, the thickness *t* of MAE films was ≈0.5 mm.

Why was the development of a new fabrication method necessary?

**Figure 3:**
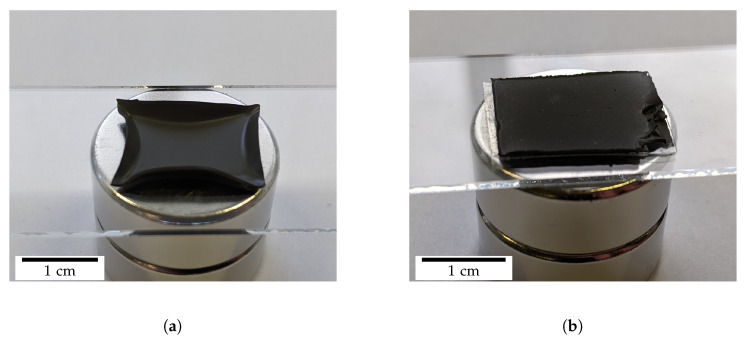
Deformation of MAE films on the permanent magnet magnetized along its height. (**a**) MAE film without plastic substrate. (**b**) MAE film on the plastic substrate.

First, the presence of a stiff substrate (carrier film) prevents deformation of MAE-based MRSs in the inhomogeneous magnetic field of a permanent magnet. This is clearly visible on Figure 3. The inhomogeneous magnetic field in the vicinity of the magnet’s edges causes the edges of non-supported MAE to curl upwards, which would obscure the camera view of the droplet.

Second, the response to the magnetic field is enhanced because the so-called particle-enriched side (PES) of the MAE film is used. Conventionally, the fabrication of MAE samples involves pouring them into a Petri dish. Therefore, each sample has two circular surfaces. The upper surface is polymerized in contact with the air, while the bottom one is polymerized in contact with the smooth surface of a Petri dish. It has been recently shown that there is a thin (≈20 µm) depletion layer under the top surface of MAEs with a reduced concentration of magnetic particles [52,57]. We will denote the “upper” side of the MAEs as the particle-depleted side (PDS). The “bottom” surface is denoted as the PES. We have observed that the PDS of the MAE film shows much higher sensitivity of wettability to the magnetic field. By employing the “upside-down” fabrication method, the PES of the MAE film becomes the upper side of the MAE-film on the plastic substrate.

A practical example for the effect of the sample side (PDS or PES) on the static contact angle (CA) can be seen in Appendix A.

In the following, the PES of MAE films was always used. The presented method allows one to reliably fabricate highly responsive MAE surfaces. A fabricated sheet of an MAE film on a plastic substrate was cut into pieces of ≈30 × 30 mm ${}^{2}$ and further processed by laser micromachining.

### 2.3. Laser Micromachining

**Figure 4:**
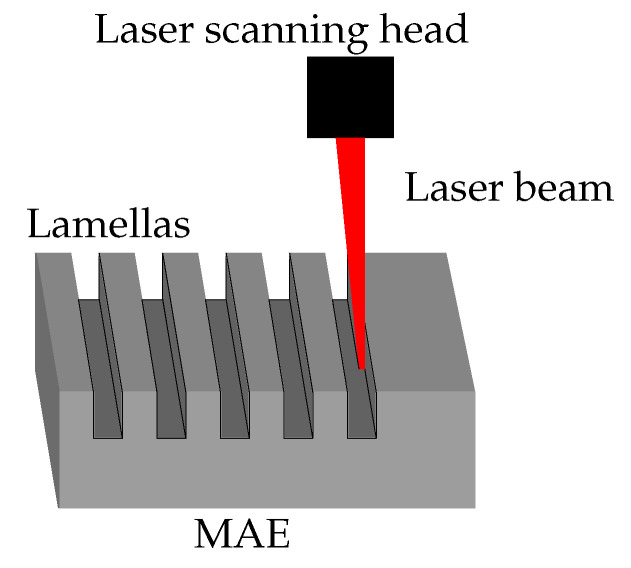
Schematic presentation of direct laser structuring.

To fabricate µm-sized structures on an MAE coating, the method of laser micromachining was used. The method is described in detail in [21]. Below, we provide a brief description of our laser processing. The principle of the micromachining process is depicted in Figure 4. A pulsed nanosecond fiber laser with a wavelength of 1064 nm, a maximum average power of 20 W, a pulse duration of 12 ns and a repetition rate of 35 kHz was used. The laser beam was guided over a preprogrammed path (speed 500 mm/s) using a scanning head (Raylase SS-IIE-10, Raylase GmbH, Weßling, Germany) with a working area 20 × 20 mm ${}^{2}$. The beam was focused onto a spot with diameter of 30 µm (measured at $1/e^{2}$ peak intensity level) using a telecentric f-theta lens (*f* = 56 mm; Ronar Smith, Singapore). The fabrication included several types of microstructures (lamellas, pillars) with different depths. The resulting structures were all slightly beveled (approximately 5°) due to the conic shape of the laser beam converging on the focusing point and due to erosion of the structure walls during material ablation.

**Figure 5:**
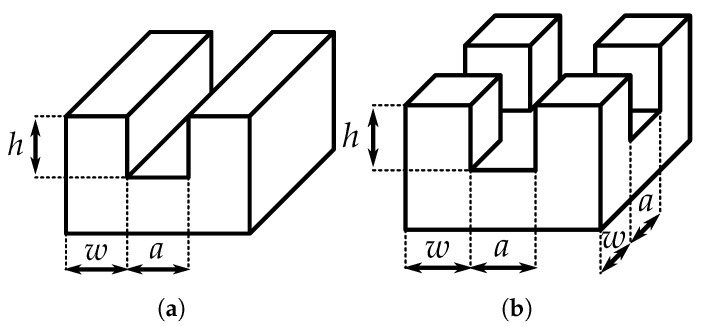
Characteristic dimensions of the surface structures. (**a**) Lamellar structures. (**b**) Pillared structures.

Figure 5 depicts the two types of surface structures produced by laser micromachining. Their characteristic dimensions are the groove depth *h*, the structure width *w* and the structure spacing *a*, which are measured at the mean height of the structures. The typical dimensions of microstructures must be significantly larger than the mean particle diameter (ca. 4.5 µm). For all fabricated structures, w≈a≈ 50 µm. The groove depth *h* was varied from ≈15 µm to ≈60 µm. Such a variation of groove depth *h* should lead to different regimes of surface wetting (Wenzel and Cassie–Baxter states). The theoretical background for different wetting states and the numerical estimate for the critical value of *h* at which there is a transition between the two wetting regimes are given in Appendix B.

Table 3 presents the notation used in the following for different settings for micromachining. The laser beam spot was scanned with a speed of 500 mm/s along the parallel lines separated by ≈15 µm, which was empirically chosen as the best option. The column “Passages” denotes the number of repetitions of laser beam transitions along the entire beam trajectory. By changing the number of passages, it was possible to vary the groove depth *h*. A single passage increased the groove depth by ≈15 µm. In the case of whole surface ablation, the laser beam was scanned over the entire surface according to the abovementioned parameters. To obtain lamellar or pillared structures, the laser processing was suppressed for the structure’s width w≈ 50 µm. This was achieved by omitting the ablation along three parallel lines. The last column in Table 3 refers to the depth of microstructures (pillars or lamellas), when only the corresponding parts of an MAE surface have been ablated. It is valid for all three MAE materials.

**Figure 6:**
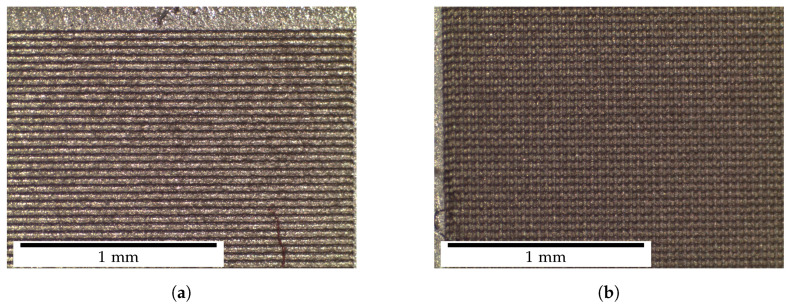
Optical microscope images of 1× laser machined surfaces of material M at 25× magnification. (**a**) Lamellar structures. (**b**) Pillared structures.

Figure 6 demonstrates two example structures created with the abovementioned processing.

It is expected that by using smaller iron filler particles, the spatial resolution of our technology can be further increased to the diameter of a focused laser beam [21]. However, it is known that for the same filler concentration, the resulting magnetomechanical response of MAEs to the magnetic field can be higher with larger particles [64].

### 2.4. Rheological Measurements

A commercial rheometer (Physica MCR 301, Anton Paar GmbH, Graz, Austria) with a plate-plate measurement cell was used to measure the shear modulus of the materials. An MAE sample without plastic substrate with a thickness of 1 mm and a diameter of 20 mm was placed into the measurement cell. To avoid slippage, a normal force of 1 N was applied and a constant oscillation frequency of ω = 10 s ${}^{-1}$ with a shear deformation of ≈1% was used.

### 2.5. Scanning Electron Microscopy Imaging

A scanning electron microscope (SEM; Helios NanoLab 650, FEI, Hillsboro, OR, USA) was used to produce highly magnified images of the samples. The measurements were carried out at the Center of Excellence for Nanoscience and Nanotechnology in Ljubljana, Slovenia. The samples were sliced into thin strips and coated with a 20 nm thick conductive layer of amorphous carbon. The imaging was performed with an acceleration voltage of 15 kV and a probe current of 0.8 nA. The images of the top and the bottom sides were taken, as well as the images of the cross-section. The iron particles can be seen as white spots inside the dark matrix.

### 2.6. Optical Profilometry

The surfaces of the samples were analyzed with a 3D optical microscope (ContourGT-K0, Bruker Corporation, Billerica, MA, USA) using a white-light interferometric objective with 5× magnification. The profilometry is based on scanning white-light interferometry, where the distance between the sample and the interferometric objective is automatically varied while the corresponding micrographs showing the vertical displacement of interference fringes are recorded. The surface data generated by the profilometer were processed and analyzed with the open source program Gwyddion [65,66]. A polynomial leveling was performed, followed by denoising with a Gaussian filter of 5 µm width.

### 2.7. Contact Angle Measurement Setup

**Figure 7:**
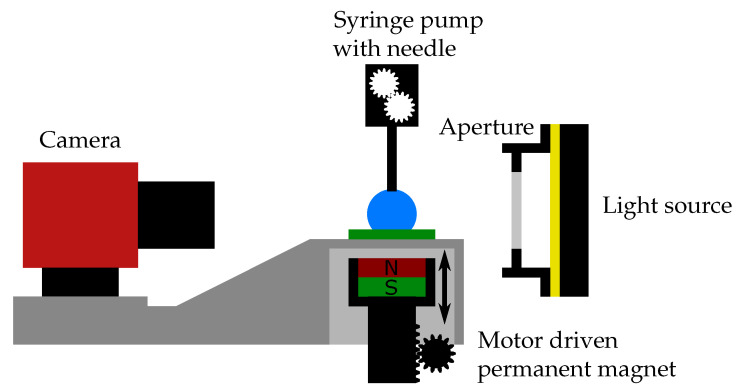
Experimental setup for contact angle measurements with a varying magnetic field.

Figure 7 presents the custom built experimental setup. Commercially available devices do not allow for the application of a variable magnetic field to the sample and typically contain ferromagnetic parts, which would interact with a magnetic field.

The setup is built on an aluminum breadboard (MBH4545M, Thorlabs Inc., Newton, NJ, USA). A camera (Alvium 1800 U-319m, Allied Vision Technologies GmbH, Stadtroda, Germany) with a macrolens (OE MC 075X, Stemmer Imaging AG, Puchheim, Germany) and a sample holder are mounted onto a rigid stand (MP100, Thorlabs Inc., Newton, NJ, USA). The light source for backlighting consist of multiple white LEDs behind a diffusor plate for homogeneous lighting. A custom 3D printed cover is used to mount an aperture in front of the light source for controlled intensity and focus depth. The custom 3D-printed sample holder is hollow. This feature allows for free vertical movement of the permanent magnet (2× stacked NdFeB cylinder magnet, diameter *ϕ* = 25 mm, height $h_{M}$ = 10 mm, grade N40). By changing the vertical position of the permanent magnet, the magnetic field in the sample can be controlled. The measurements were performed on the sample’s surface in the vicinity of the symmetry axis of the permanent magnet, where the magnetic field can be considered uniform and directed perpendicular to the sample’s surface. The permanent magnet created a magnetic flux density of B≈ 400 mT on the sample’s upper surface.

The magnet is driven by a linear stage (Movtec Wacht GmbH L60, Pforzheim, Germany), which allows for fine control of the distance. A gaussmeter (Model 450, Lake Shore Cryotronics Inc., Westerville, OH, USA) with a Hall sensor head (HMMT-6J04-VR, Lake Shore Cryotronics Inc., Westerville, OH, USA) was used to map the magnet distance to the magnetic field at the sample position for reference in the measurements.

The dispensing of deionized water (specific electric conductivity σ≈ 3 µS/cm) was controlled by a syringe pump (Microliter OEM Syringe Pump, Harvard Apparatus, Holliston, MA, USA) using a Hamilton Gastight Syringe 1ml and a Braun Sterican® 27 G × 1” blunt tip. The pump was attached to a vertical stage (Standa 180544, Vilnius, Lithuania) with a 90° bracket to allow for fine height adjustment of the needle tip.

### 2.8. Static Contact Angle Measurement Protocol and Evaluation

The static apparent contact angle is determined using the following measurement protocol, according to recommendations in [67]. The measurements were performed in standard laboratory conditions (ambient temperature ϑ=(23±1) °C, relative humidity RH=(50±10)%). First the magnetic field calibration is performed without a sample by measuring the magnetic flux density at the upper surface of the sample holder for different magnet positions in the vertical direction. Then, the sample is checked for damages and any dust or debris are removed with a piece of adhesive tape before it is placed onto the upper surface of the sample holder. Following this, the desired magnetic field is set by moving the linear stage in position according to the calibration data. Subsequently a droplet of the desired volume (4 µL) is dispensed from the pump and then lowered towards the sample surface. The needle is then extracted from the droplet. Note that the droplet is positioned in the vicinity of the vertical axis of symmetry of the permanent magnet, where the magnetic field can be considered to be vertical and uniform. The droplet is then left to rest for at least 30 s to allow the system to come to equilibrium. During this time, the exposure and light intensity are adjusted to yield optimal image quality and the operator determines the contact line of the droplet.

**Figure 8:**
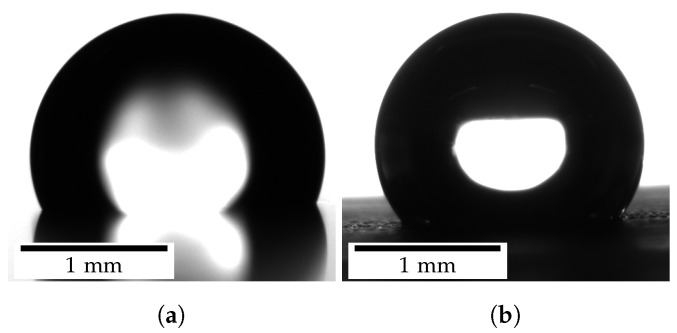
Images of water droplets on MAE surfaces as recorded by the measurement setup. (**a**) Droplet on reflective MAE surface outside magnetic field; reflection and contact line are clearly visible. (**b**) Droplet on matt MAE surface inside magnetic field; no reflection is visible and the contact line is difficult to determine.

The contact line is determined at a higher exposure time (8.0 ms) if necessary and the droplet contour measurement is performed at a lower exposure time (2.0 ms). Such an increase in the exposure time is often required because the MAE surface becomes matte (less reflective, see Figure 8) due to the significantly grown SR, which impedes the identification of the contact line.

After the equilibrium state is reached, an image is taken and the droplet contact angle is determined by analyzing its shape. The contour of the droplet is obtained by using Canny filtering and edge detection algorithms. This contour is split at the apex of the droplet into two separate sets of points, representing the left and right side of the droplet. For each set, an ellipse is fitted using the OpenCV library [68], which implements the “LIN: Algebraic Distance” algorithm mentioned in [69]. Then, at the intersection of the contact line and ellipse, the tangent to the ellipse is determined. The angle of the tangent to the contact line is the CA of the droplet. The operator can visually determine the quality of droplet detection and fit and intervene if other measures need to be taken. Finally, the droplet is removed with compressed air. The magnetic field can now be adjusted and a new droplet dispensed. To avoid possible hysteresis, the magnetic field is only increased during concurrent measurements and the sample is left to rest at least 24 h before another measurement at a lower magnetic field is conducted.

### 2.9. Statistical Analysis

Statistical analysis for CA measurements was performed using Python with its data analysis and manipulation module “pandas” and statistics module “scipy.stats”. All other measurements were evaluated with Microsoft Excel. All results were statistically significant with p≤0.05 and a sample size (n) of at least 5. The data is summarized and plotted as the mean, with error bars representing the 95% confidence interval.

## 3. Results

### 3.1. Characterization of Unstructured MAE Surfaces

**Figure 9:**
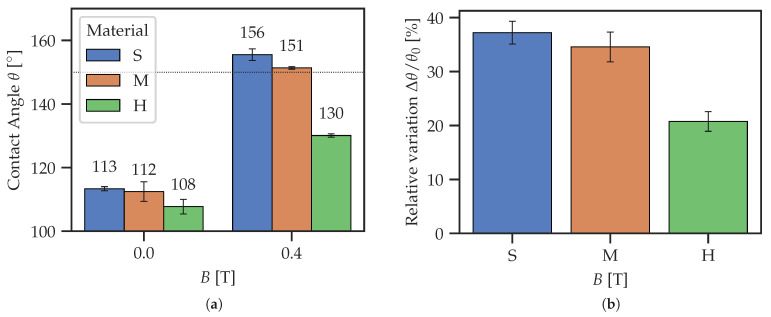
Comparison of magnetic field effects for MAE films of different stiffnesses. (**a**) Switching of CA with magnetic field. (**b**) Relative magnetic field-induced variation of CA.

The objective of this subsection is to compare the wetting properties of unstructured MAE films with different stiffnesses (Figure 9). The maximum CA of (156 ± 2)° is achieved for the softest MAE material. This result is comparable with the highest reported value of (163 ± 2)° [43], which was achieved using an anisotropic (i.e., cured in the presence of magnetic field) MAE sample in a higher magnetic field of 0.6 T. It confirms that significant sensitivity of the CA to a magnetic field can also be achieved in ultra-soft MAE samples cured in the absence of a magnetic field. In the following figures, the horizontal dashed line θ = 150° serves to indicate the superhydrophobicity θ > 150°.

The relative change in the CA (≈35%), which is defined as

(1) $$\Delta \theta /\theta_{0}=\frac{\theta (0.4\;T)-\theta (0.0\;T)}{\theta (0.0\;T)}\;\cdot \;100\%,$$

is very similar for the softer materials S and M.

On the other hand, the stiffer material H demonstrates much lower responsiveness to the magnetic field. This agrees well with the previous explanation [43] that a soft polymer matrix promotes the restructuring of the filler below and at the surface of MAE coatings. Furthermore, we suggest that the application of a magnetic field of 0.4 T leads to the Cassie–Baxter regime for materials S and M, while the material H stays in the Wenzel regime. Such a change in the wetting regime is indicated by the appearance of the total internal reflection of light on the contact area, visible to the naked eye. This total internal reflection is caused by the air pockets below the droplet, which is typical for the CB regime.

### 3.2. Effect of Laser Surface Ablation on Contact Angle

**Figure 10:**
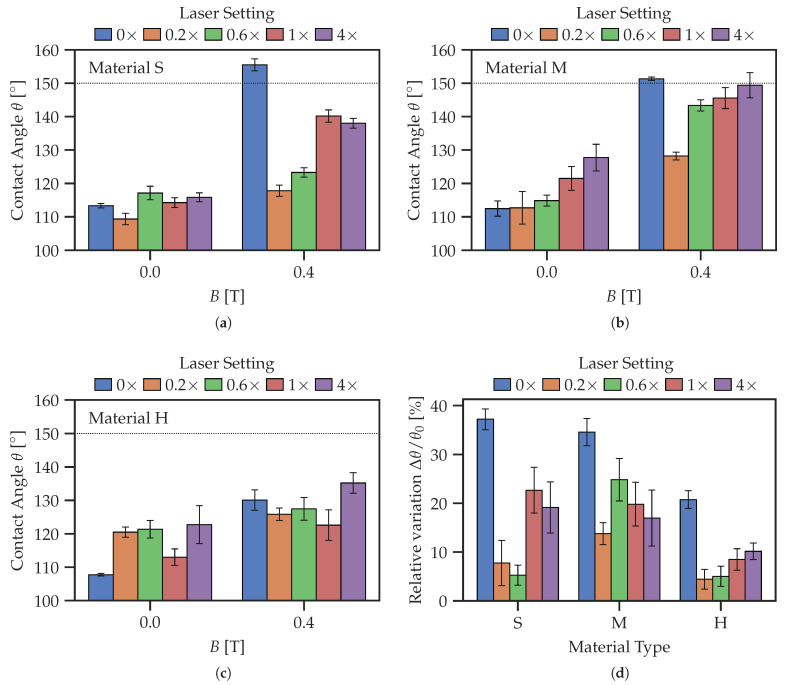
Comparison of contact angles on laser treated MAE surfaces for different laser power settings. (**a**) Material S; (**b**) Material M; (**c**) Material H. (**d**) Relative change in the contact angle $\Delta \theta /\theta_{0}$.

To analyze the specific effects of laser surface ablation on the CA, full areas (5 × 5 mm ${}^{2}$) were processed by the laser with different settings, as given in Table 3. Figure 10 summarizes the CAs measured. Figure 10d compares the relative changes in the CA expressed as $\Delta \theta /\theta_{0}$ between passive and active states. Both figures show a significant degradation of responsiveness of the CA to the magnetic field due to laser ablation. The unprocessed surfaces (0×) show the highest CA in the magnetic field, while also possessing a low CA in the initial state, resulting in the highest relative change. For other ablation settings, there seems to be a local maximum of $\Delta \theta /\theta_{0}$ between the 0.6× and the 1× settings. We speculate that the existence of this local maximum of $\Delta \theta /\theta_{0}$ can be explained by a competition between increasing surface hardness (thermal sealing of the surface) and increased SR of treated surfaces. This effect must be investigated in more detail in the future.

### 3.3. Effects of Surface Structuring

**Figure 11:**
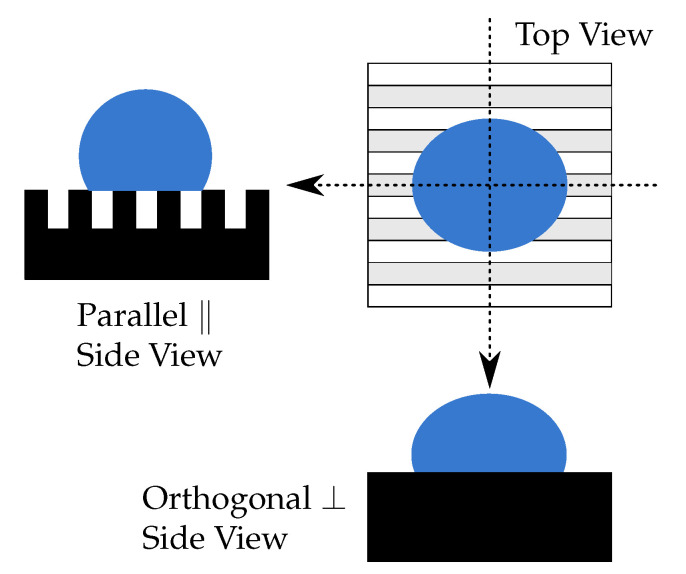
Different viewing angles for CA measurements on lamellar MAE surfaces. In the ‖-direction, the line of sight is parallel to the surface structures (long side of lamellas). In the ⊥-direction, the viewing axis is orthogonal to the long side of the lamellas.

The roughness geometry of lamellar structures is not isotropic [70,71]. Therefore, they show intriguing directional behavior, namely, the apparent CA is no longer uniform along the contact line. As Figure 11 demonstrates, the CA was measured in two different directions. In the “parallel“ (‖) direction, the lamellas point towards the camera, revealing an image with visible ridges. In the “orthogonal“ (⊥) direction, the lamellas form an angle of 90°with the viewing direction of the camera. A similar phenomenon has been previously investigated for other materials: pure PDMS in [71], silicon in [70] and MgF ${}_{2}$ in [72].

Pillared structures have a higher symmetry (the cross section is a square); therefore, the CAs in the ‖- and ⊥- directions (parallel to the square sides) are the same.

**Figure 12:**
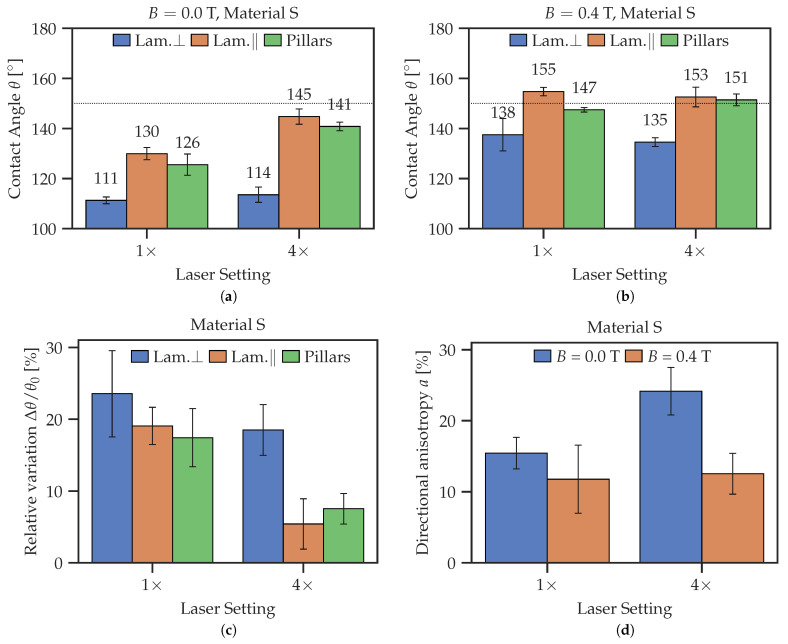
Comparison of CAs on differently structured MAE surfaces from material S with 1× and 4× laser passages. (**a**) Comparison at *B* = 0.0 T. (**b**) Comparison at *B* = 0.4 T. (**c**) Relative change of CA $\Delta \theta /\theta_{0}$ versus magnetic field. (**d**) Directional anisotropy of CA on lamellas with respect to the viewing direction.

Figure 12 demonstrates how the CA on the same lamellar structure differs when changing the viewing angle. In the ⊥-direction, lamellas show a similar behavior to the unstructured surface, whereas in the ‖-direction, lamellas (and pillars in general) have a high initial CA.

Figure 12c shows the relative change of CA. Lamellas in ⊥-direction express the highest sensitivity to the magnetic field. Increasing the number of laser passages diminishes the responsiveness to the magnetic field, as described above in Section 3.2. Interestingly, the values of the CAs on pillared MAE surface are mostly between the CAs of lamellas in the ‖-and ⊥-direction, more similar to the lamellas in the ‖-direction. As must be expected, it is much easier to reach superhydrophobicity on structured surfaces due to the Cassie–Baxter regime.

Figure 12d highlights the change of CA on lamellas caused by anisotropy between the ‖- and ⊥-directions, which is defined as

(2) $$a=2\frac{\theta_{\|}-\theta_{⊥}}{\theta_{\|}+\theta_{⊥}}\;\cdot \;100\%.$$

The largest anisotropy of the CA was detected for the 4× laser setting without a magnetic field. It is also clearly visible that applying a magnetic field decreases this difference for both laser settings.

**Figure 13:**
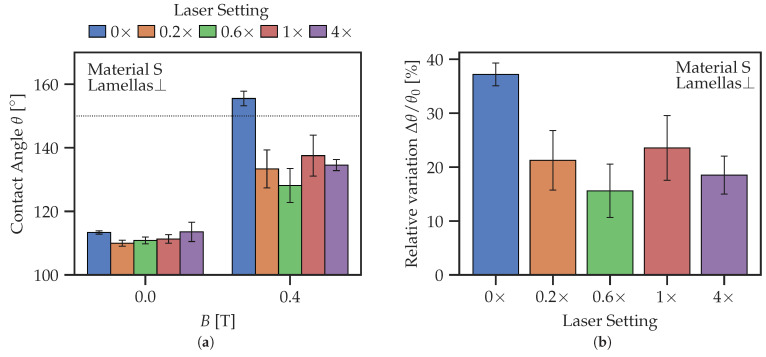
Contact angles on lamellar structures from material S in the ⊥-direction for different laser settings. (**a**) Absolute value of CA. (**b**) Relative change in CA caused by magnetic field.

Figure 13a shows the dependence of CA on the laser settings in more detail. Similar to the full area laser processing (Section 3.2), the unprocessed surface has the highest responsiveness, while the laser processing reduces the dynamic range of CA. A local maximum of the relative change in CA is observed for the 0.6× or 1× laser processing regimes, similar to the observation reported above.

## 4. Discussion

The new fabrication method of MAE films on PET substrates makes use of the enhanced response of the PES to magnetic fields for switching of the CA.

The laser treatment of the entire MAE surface causes it to be less responsive to the change in magnetic field. This is shown by Figure 10d, where the relative change in CA is the highest for the unprocessed surface. As far as the responsiveness of laser-ablated MAE surfaces to magnetic fields is concerned, there seems to be an optimum at one passage (1× regime) of the laser beam at full power. This can be explained by the counteracting effects of the increased SR and increased surface hardness on the resulting CA.

**Figure 14:**
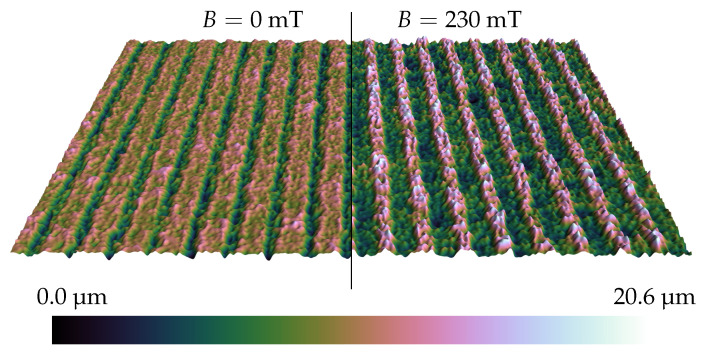
Effect of a magnetic field on surface morphology of a lamellar MAE structure from material M (left: *B* = 0 T | right: B≈ 230 mT). The vertical scale is exaggerated for a better visualization. Shown area is 1.72 × 1.29 mm ${}^{2}$. Laser setting is 1×.

Direct laser structuring of MAE surfaces changes the wetting behavior drastically (Figure 12). The initial and maximum contact angles increase; the material may even become superhydrophobic in the magnetic field. We attribute this change in the CA to the deformation of microstructures in an external magnetic field, similar to the elongation (magnetostriction) of MAE bodies along an external magnetic field; cf. [73,74,75,76]. This hypothesis is supported by Figure 14, which shows optical profilometry results for a lamellar MAE structure. The changes in surface morphology between passive and active states are clearly visible. The magnetic field seems to produce the following effects: roughening of the top surface and elongation and thinning of ridge-like structures. Both effects would cause the CA to increase, according to the hierarchical form of the Cassie–Baxter Equation (A4).

The lamellar structures show interesting directional properties (Figure 11). Contact angles measured in two perpendicular directions exhibit very different wetting behaviors, as seen in Figure 12. In the ⊥-direction, the lamellas show a wetting response similar to the unstructured surfaces, namely, a low (≈110°) initial CA and high responsiveness to the magnetic field. The 4× surface is less responsive to the magnetic field than the 1× surface, which could be caused by the hardening of the surface due to a thicker heat-affected layer caused by the laser ablation. In the ‖-direction, the CA is very high (≈145°) in the absence of a magnetic field, but shows almost no response to the magnetic field.

As previously obtained for other materials in [70,71,72], the droplet shape is no longer spherical, but elongated in the direction of the lamellas due to directional anisotropy of the wetting behavior. Furthermore, the likely roughening of the top of the lamella ridges will increase the CA according to Equation (A4). However, an increase in the SR of the top of lamella ridges affects primarily the wetting in the ‖-direction, while in the ⊥-direction, the effect of large gaps between lamella ridges dominates.

The results for pillared structures in Figure 12a,c are not affected by the anisotropy, as they show similar geometry along both axes (square cross-section in lateral directions). In further experiments, it would be interesting to destroy this symmetry and to investigate wetting of MAE pillars with non-squared cross-sections.

Deeper microstructures cause higher contact angles in the absence of a magnetic field, although in the CB regime, the structure height should not affect the CA at all. The effect can be attributed to the thinning of the top part of the structures, as the laser beam is slightly cone shaped (see Figure 4) and produces structures that are thinner at the top.

Figure 12 shows that the CA of the pillared structures is not as sensitive to the magnetic field as the lamellas in the ⊥-direction and is roughly the same as for lamellas in the ‖-direction. This could be expected, as those geometries both show similar gaps.

**Figure 15:**
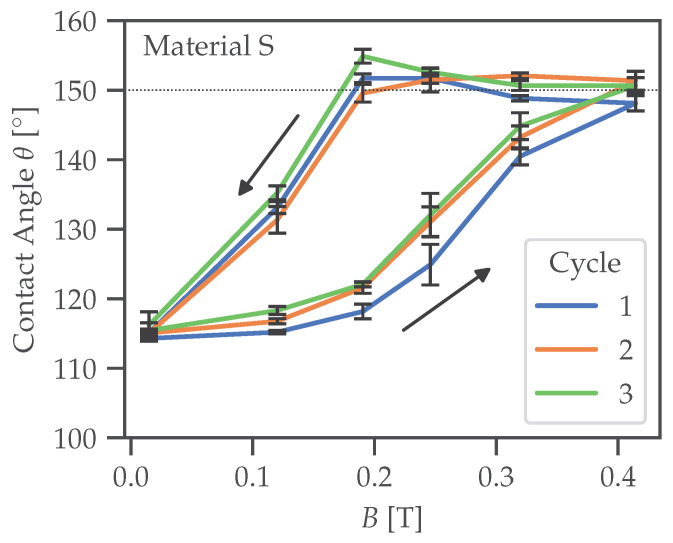
Magnetic-field dependence of the static contact angle for an unstructured MAE sample. Three magnetization cycles are shown. The arrows designate the direction of magnetic-field change.

Presented results demonstrate for the first time that it is possible to fabricate microstructured MRSs on the basis of MAE elastomers for effective control of their wettability. The results are promising. However, further research is required to investigate the wettability on MAE coatings and to gain a deeper insight into the underlying physical effects. First, in the present paper as well as in the previous publications [43,44], the static CA was considered. In following work, we intend to investigate the advancing and receding contact angles (i.e., CA hysteresis) and their dependence on the applied magnetic field. Second, it is well known that the magnetomechanical response of MAEs shows a pronounced hysteresis behavior with respect to the applied magnetic field [59,76], even for soft-magnetic inclusions, which is related to the hysteresis of the restructuring of the filling particles [59,77,78,79,80]. It is expected that static, advancing and receding contact angles display such hysteresis with respect to the external magnetic field. In the present paper, we have avoided this issue by considering only the increase in the magnetic field and the comparison of passive and active states. As an example, Figure 15 presents the measurement results for the dependence of static CA on the magnetization history for an unstructured MAE sample. It is seen that the initial (increasing magnetic field) curve significantly differs from the subsequent cycles. As explained in [59], it can be expected that during the first cycle, principal restructuring of the filler takes place. Initially, this results in major changes to the particles’ microstructure, whereas further changes are minor. Third, it will be interesting to investigate the phenomena of dynamic wetting on unstructured and microstructured MAE-based MRSs, in particular, their dependence on the contact line velocity. We believe that investigations of the dynamic wetting phenomena of MAE-based MRSs are just in their initial stage, in particular, because of the lack of systematic experimental investigations and theoretical models.

Further attention should be paid to the fabrication of an MAE surface that is initially hydrophilic (θ<90°). In this case, a magnetic field might provide a transition to a highly hydrophobic state. The initial hydrophilic property of MAE surfaces can be possibly achieved by surface modifications such as coating or irradiation; see, e.g., [81,82].

Since no information is available in the literature regarding the effects of laser ablation on MAE surfaces, it would be interesting to investigate how the elemental composition of the surface changes after laser treatment, e.g., by the SEM-EDS (energy dispersive spectroscopy) method.

## 5. Conclusions

To summarize, we have investigated the static wetting behavior of unstructured and structured MAE coatings on plastic substrates. The following conclusions can be made from our work.

- A new method of fabricating large area, highly sensitive MAE coatings on plastic substrates creates experimental prerequisites for developing magnetically responsive smart surfaces.
- Our preliminary work has shown that sedimentation of filling particles does have significant influence on the sensitivity of static CA to changes in magnetic field. Usage of the PES allows one to achieve large changes of the CA from (113 ± 1)° in the absence of the field to (156 ± 2)° in the magnetic field of 400 mT. These values compare well with previously published values [43], although no crosslinking in the external magnetic field was required.
- Laser ablation of the entire MAE surface causes the CA to react more weakly to magnetic fields due to surface degradation. However, this reduction of sensitivity to magnetic fields is not monotonic with the increasing value of total laser power used for surface treatment. We observed that the maximum responsiveness of CA to magnetic field is achieved either for the 0.6× or 1× regimes of laser processing.
- Direct laser structuring has a significant effect on the wettability of MAE surfaces and their response to magnetic field. In general, the CA becomes larger and superhydrophobicity can be achieved (θ>150°). For the lamellar structures, a clearly pronounced anisotropy of the CA with respect to the viewing direction is observed. The highest relative response of CA to magnetic field is obtained for lamellar structures from the softest material in the ⊥-direction, although the absolute change in CA for the ⊥- and ‖-directions of roughly 26° is similar.

## Figures and Tables

**Table 1:** Proportions of individual components for fabrication of MAE samples in wt.%.

Material	CIP	VS 100,000	MV 2000	AK10	Modifier	CL 210	Inhibitor	Catalyst
Soft (S)	74.625	7.128	1.283	16.713	0.025	0.114	0.027	0.085
Medium (M)	74.927	7.026	1.282	16.505	0.025	0.117	0.030	0.088
Hard (H)	74.825	7.063	1.272	16.559	0.025	0.130	0.029	0.097

**Table 2:** Viscoelastic properties of MAE materials used.

Material	Shear Storage Modulus *G*’ [Pa]	Shear Loss Modulus *G*” [Pa]
Soft (S)	8717 ± 71	2168 ± 5
Medium (M)	14,516 ± 69	2775 ± 5
Hard (H)	22,085 ± 198	2188 ± 28

**Table 3:** Laser power settings nomenclature.

Setting	Passages	Laser Power [W]	Structure Depth *h* [ µm]
0×	Unprocessed Surface	-	0
0.2×	1	4	≈3
0.6×	1	12	≈9
1×	1	20	≈15
4×	4	20	≈60

**Figure A1:**
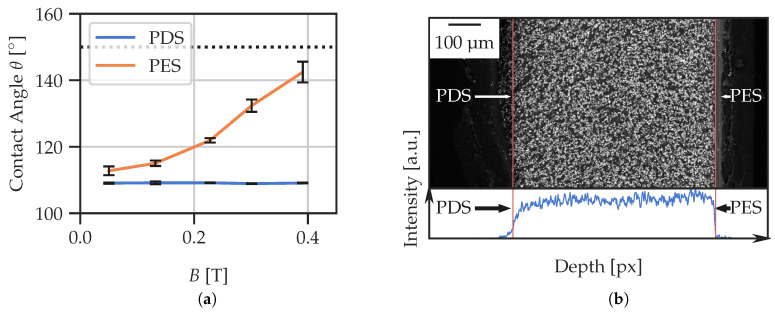
Example of differences between PDS and PES. (**a**) Comparison of the effect of magnetic field on CA for the PDS and the PES of an exemplary MAE sample. (**b**) SEM image of the cross-section of an MAE sample.

**Figure A2:**
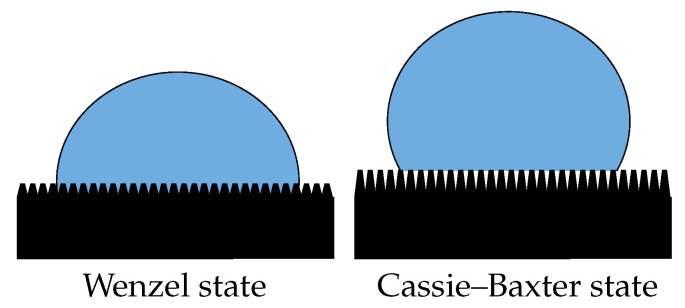
Droplet contact line for Wenzel and Cassie–Baxter wetting regimes. In the Wenzel regime, the droplet is in full contact with the entire surface; in the Cassie–Baxter regime, it only wets a fraction of the total surface.

## Data Availability

The data supporting the findings of this paper can be obtained from the corresponding author, R.K., upon reasonable request.

## Abbreviations

The following abbreviations are used in this manuscript:

CA	Contact angle
CB	Cassie–Baxter
CIP	Carbonyl iron powder
CL	Crosslinker
MAE	Magnetoactive elastomer
MRS	Magneto-responsive surface
PDMS	Polydimethylsiloxane
PDS	Particle-depleted side
PES	Particle-enriched side
PET	Polyethylene terephthalate
PS	Polystyrene
SEM(-EDS)	Scanning electron microscopy (with energy dispersive spectroscopy)
SR	Surface roughness
W	Wenzel
⊥	Orthogonal to the direction of lamellas
‖	Parallel to the direction of lamellas

## Appendix A

The purpose of this appendix is to illustrate the differences between the PDS and PES. As an example, Figure A1a compares the static apparent contact angle (CA) θ of the PDS and PES of an MAE film. It is seen that the CA of the PES responds very well to the applied magnetic field, while the PDS shows almost no response. As explained above, this effect is attributed to some sedimentation of filling particles during the process of fabrication. Such a sedimentation is illustrated in Figure A1b, where the SEM (scanning electron microscope) image of an MAE film cross-section is shown, and the blue line presents the sum of intensity of pixels along the vertical lines. The white spots are the iron particles dispersed in the polymer matrix. The distribution of these particles is much thinner on the far left side (top of the sample) than on the inside or the right (bottom) side. There is also a small region on the far right with the highest particle concentration.

## Appendix B

The purpose of this appendix is to briefly recall the theoretical background of wetting on rough solid surfaces and to estimate the critical groove depth $h_{c}$ where a transition between Wenzel (W) and Cassie–Baxter (CB) wetting regimes takes places (Figure A2).

Consider first the Wenzel model, when the liquid is in total contact with the surface. The apparent CA $\theta_{W}$ of a liquid on a chemically homogeneous surface is directly related to its roughness parameter r≥1, which is the total surface area divided by the projected area,

(A1) $$\cos\theta_{W}=r\frac{\gamma_{SA}-\gamma_{SL}}{\gamma }=r\cos\theta_{Y},$$

where $\theta_{Y}$ is Young’s CA (on a smooth surface); $\gamma_{SA}$, $\gamma_{SL}$ and γ are the surface tensions at the solid/air (vapor), solid/liquid and liquid/air interfaces, respectively [83]. Changing the roughness parameter *r* of the surface will modify the apparent CA. For hydrophilic surfaces where $\theta_{Y}<90$°, increasing the roughness will make the surface more hydrophilic. Likewise, for hydrophobic surfaces $(\theta_{Y}>$90°), an increase in the roughness makes the surface more hydrophobic [83,84]. The latter effect occurs in this work, because the used material is hydrophobic and the magnetic field increases the SR.

The Cassie–Baxter (CB) Equation (A2) is used to explain wetting on mixed surfaces, where one part of the surface has CA $\theta_{1}$ and the other part has CA $\theta_{2}$. The surface types each occupy a fraction of the total area $f_{1}$ and $f_{2}$, respectively. According to [83], the apparent CA $\theta_{CB}$ of a droplet on such a surface can be described by the CB equation

(A2) $$\cos\theta_{CB}=f_{1}\cos\theta_{1}+f_{2}\cos\theta_{2}.$$

If the surface has sufficiently high-aspect-ratio structures, the Wenzel regime may no longer be used to explain the wetting phenomena, because the gaseous phase creates pockets between the liquor and solid phase due to the surface tension of a liquid, and the droplet is not in total contact with the surface. If the surface consists of high-aspect structures, the water cannot penetrate into the grooves, meaning the droplet is now partially supported by the solid and the air $\theta_{2}=\pi$ with $f_{1}=1-f_{2}$. From this, it follows that:

(A3) $$\cos\theta_{CB}=f_{1}\cos\theta_{Y}+f_{1}-1.$$

The part of the surface wetted by the droplet in the CB regime is not usually perfectly flat. If the effect of the wetted surface roughness affects the wetting significantly and the result deviates from the CB prediction, one can assume a hierarchical wetting scenario [83,85,86]. In this case, the roughness of the wetted portion is taken into account by the addition of the roughness factor *r* into the CB equation:

(A4) $$\cos\theta_{CB}=rf_{1}\cos\theta_{Y}+f_{1}-1.$$

It is well known that surfaces can obtain hydrophobic properties by structuring them in a certain way using molding, additive processes or laser micromachining [21,87,88,89,90]. However, these papers did not deal with soft MAEs as in the present paper.

For pillared structures, the transition from the Wenzel to Cassie–Baxter regimes can be estimated using the following expression for the critical height $h_{c}$ [91]:

(A5) $$h_{c}=-\frac{a}{2}\left(1+\sec\theta_{Y}\right).$$

If $h<h_{c}$, the Wenzel regime must be expected. If $h>h_{c}$, the Cassie–Baxter regime must be expected.

For the used groove width a≈ 50 µm and Young’s CA of water on PDMS $\theta_{Y}\approx$ 110°, an approximate critical height $h_{c}\approx$ 34 µm is obtained. Therefore, the fabricated structures had heights varying from ≈3 µm to ≈60 µm, so both the Wenzel and Cassie–Baxter regimes for pillared structures can be expected.

Similarly, a gradual change from Wenzel to Cassie–Baxter wetting regimes can be anticipated with increasing lamella height *h* in the absence of a magnetic field.
